# Water Availability and Leaf Microstructures Jointly Regulate Dew Absorption in Plants with Different Ecotypes

**DOI:** 10.3390/plants15030503

**Published:** 2026-02-05

**Authors:** Qilong Qiu, Yingying Xu, Jiahe Miao, Yunze Zhao, Hong Jiang, Yingtan Wu, Jinyue Ma

**Affiliations:** 1 Key Laboratory of Songliao Aquatic Environment, Ministry of Education, Jilin Jianzhu University, Changchun 130118, China; 19816645697@163.com (Q.Q.); 15948778676@163.com (Y.Z.); 15604434961@163.com (J.M.); 2 Fujian Engineering and Research Center of Rural Sewage Treatment and Water Safety, Xiamen University of Technology, Xiamen 361024, China; miaojiahe@xmut.edu.cn; 3 Jilin Science and Technology Museum, Changchun 130022, China; 17790003078@163.com; 4 School of International Exchange, Jilin Jianzhu University, Changchun 130118, China; 17704383066@163.com

**Keywords:** foliar water uptake, water absorption, water transport, stable isotope tracer experiment, leaf microstructure

## Abstract

Dew formation occurs frequently and in substantial amounts, serving as an important water source with significant ecological implications for plant growth. Although previous studies have demonstrated that dew can supplement leaf water, quantitative evidence of leaf dew absorption under different dew intensities remains limited. In this study, a stable isotope tracer experiment was conducted to quantify dew absorption under varying dew amounts and to analyze absorption rates and influencing factors across different plant species. Results showed that all four species were capable of absorbing dew, mainly due to specialized leaf surface morphology and microstructures. At a dew intensity of 0.1 mm, *Tillandsia ionantha*, whose leaves are densely covered with shield-like trichomes, exhibited an extremely high dew absorption rate of 92%. In contrast, the leaf surface of purple shamrock (*Oxalis triangularis* ‘Purpurea’) is covered with abundant hydrophobic trichomes that strongly restrict dew entry, resulting in a very low absorption rate of only 1.43%. Dew absorption varied markedly among species under different dew amounts. Under dew intensities of 0.1, 0.2, and 0.3 mm, *T. ionantha* showed consistently high absorption rates of 92%, 89.60%, and 71.74%, respectively, whereas *Epipremnum aureum* exhibited much lower rates of 3.72%, 6.15%, and 2.45%. Moreover, under a dew intensity of 0.2 mm, dew absorbed by *E. aureum* leaves could be transported to the roots, indicating internal redistribution of foliar-absorbed water. Overall, dew represents an important supplementary water source for plants, and interspecific differences in leaf surface morphology and microstructures lead to substantial variation in dew absorption capacity. These findings provide experimental evidence for understanding species-specific strategies of dew utilization and have implications for the efficient use of dew as a water resource.

## 1. Introduction

Water vapor in the air condenses into droplets on the leaf surface once the leaf surface temperature falls below the dew point, thereby wetting the leaf and enabling subsequent water absorption and utilization by the plant [1,2]. The role of dew as an important water source for plant growth has been confirmed by numerous researchers [3,4]. For example, dew absorbed by leaves can directly boost *Populus euphratica*’s water status, enhance photosynthesis, and improve drought resistance, acting as a key water source in arid ecosystems [5]. In arid regions, *Leymus chinensis* and *Agropyron cristatum* absorbed dew under drought, boosting leaf water content and potential, and enhancing photosynthesis, growth, and leaf structure [6]. Studies on the Mongolian Plateau found that C_4_ herbaceous plants have higher dew use efficiency than drought-tolerant C_3_ species, with dew enhancing leaf water content, photosynthesis, root biomass, and total biomass under drought [7]. These research examples show that plant species differ markedly in their ability to absorb dew. In previous studies exploring plant utilization of dew, most research has assessed the importance of dew absorption by plants based on changes in water potential and biomass following dew uptake [8,9]. For instance, in field and greenhouse experiments with maize in semi-arid regions, by monitoring changes in leaf water potential before and after dew wetting, it was found that dew absorption helps increase leaf water potential under water stress, improves photosynthesis, and alleviates reductions in photosynthetic rate [10]. Other studies have compared leaf weight before and after dew events, finding that dew absorption can enhance plant water status and improve drought resistance [8]. However, based on the studies above, it is difficult to directly determine the source of water utilized by plants (such as dew, fog, or irrigation). This has left scientists with questions about the specific role of dew in plant growth, as well as the mechanisms and pathways of its absorption. By combining stable isotope (δ^18^O, δ^2^H) tracing techniques, the relative contribution of dew to plant water can be accurately identified, allowing quantitative assessment of dew absorption by different plant leaves, which is of great significance for understanding the ecological role of dew in plants.

Related studies have also used isotope techniques to verify that plant leaves are capable of absorbing dew. For example, when simulating dew spraying, the proportions of dew absorbed by the leaves of four shrub species—*Artemisia ordosica*, *Hedysarum mongolicum*, *Salix psammophila*, and *Caragana korshinskii*—were 20.00 ± 7.00%, 8.40 ± 6.40%, 7.8 ± 1.90%, and 3.10 ± 2.30%, respectively [11]. Under indoor potted conditions, experiments on *Zygocactus truncatus*, *Chlorophytum comosum*, and *Juniperus formosana* showed that the amount of dew absorbed by *Z. truncatus* leaves (25.96 ± 2.69%–34.81 ± 4.61%) was significantly higher than that of *C. comosum* (20.50 ± 1.89%–23.39 ± 4.35%) and *J. formosana* (6.26 ± 0.69%–11.95 ± 1.35%) [12]. A study applied labeled stable isotope water to three plant species in the Negev Desert (either to leaves/stems or to soil) and tracked isotope distribution in roots and stems. It was found that different species can absorb dew to varying degrees through leaves or roots, with corresponding isotope changes detected in roots or stems. This study confirmed the dew utilization ability of some desert plants, but the method was not sufficiently direct to visually demonstrate the subsequent movement of absorbed dew within the plant [13]. Moreover, due to the diversity of plant species and the multiple pathways of water absorption, this approach is insufficient to accurately reveal the movement of dew within plants. This study employed stable isotope tracing to analyze the proportion of dew transported from leaves to roots after foliar water uptake, thereby directly revealing the movement of water following leaf absorption. This approach is therefore critical for elucidating the mechanisms of foliar water uptake, identifying key influencing factors, and improving plant efficiency in utilizing dew resources.

Multiple studies have shown that dew, as a common non-precipitation water source, varies substantially depending on regional and climatic conditions. For example, large dew collectors in India observed a daily dew amount of approximately 0.086 mm day^−1^ [14], while in tropical and Mediterranean climate regions, dew amounts of about 0.10 and 0.17 mm/day were recorded, respectively; in observations in Croatia, the dew amount was around 0.081 mm day^−1^ [15]. The observed daily dew intensity across different ecosystems (wetlands, farmland, and urban areas) ranges approximately from 0.001 mm to 0.315 mm, with averages of about 0.125 ± 0.069 mm in wetlands, 0.061 ± 0.026 mm in farmland, and 0.028 ± 0.009 mm in urban areas [16]. However, considering variations in geographic location, climate (e.g., humid vs. arid), and environmental factors, studies have found that the dew intensity in different regions typically ranges from 0.1 to 0.3 mm day^−1^ [17]. Therefore, this study selected dew intensities of 0.1, 0.2, and 0.3 mm to simulate scenarios ranging from extremely scarce to abundant dew, systematically investigating the characteristics of plant dew absorption, with the aim of verifying that dew can provide supplemental water to plant leaves.

The overall aim of this study is to elucidate the mechanisms by which plants absorb dew through their leaves and to explore the differences in this process across plant species and microstructural leaf contexts, as well as its ecological significance. To achieve this aim, this study selects *Tillandsia ionantha*, *Epipremnum aureum*, *Oryza sativa*, and *Oxalis triangularis* ‘Purpurea’ as research subjects, allowing a multidimensional investigation of plants’ dew absorption capacity through leaf, root, and stem structures as well as physiological traits. These four plants exhibit distinct characteristics in water-use strategies, leaf morphology, stomatal distribution, and underground water storage structures, representing epiphytic, succulent, crop, and perennial herbaceous plants. They provide insights into different ecological adaptation strategies and water acquisition mechanisms, offering important references for understanding plant adaptation to environmental water availability. This study aimed to, under controlled greenhouse conditions using stable isotope tracing: (1) quantitatively evaluate foliar dew uptake across different plant species; (2) compare differences in dew absorption efficiency among plants subjected to varying dew intensity treatments; and (3) integrate leaf microstructural characteristics to elucidate interspecific differences in foliar dew absorption capacity and the internal transport of absorbed water within plants.

## 2. Results

### 2.1. Dew Disappearance Time on Plant Leaves

**Figure 1:**
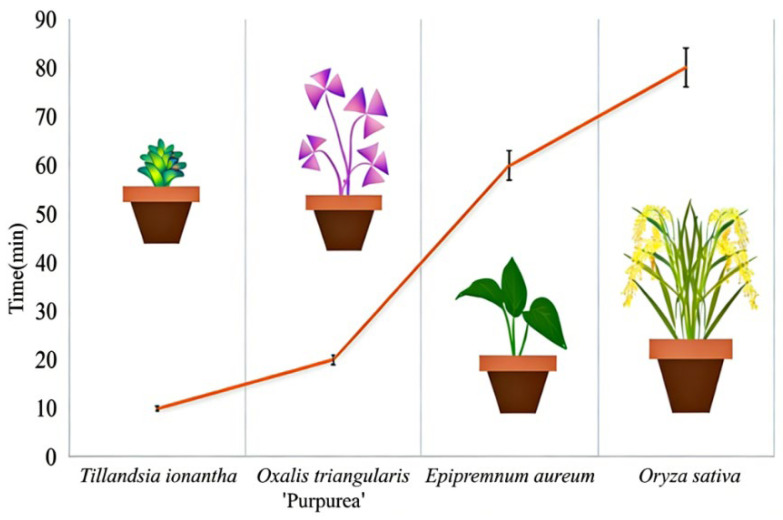
Comparison of dew disappearance time from leaves of four plant species under the same growth conditions and dew intensity (0.1 mm). Error bars indicate standard deviation (*n* = 6).

In this study, under the same growth conditions, a high-definition camera was used to continuously record the disappearance time of dew on the leaves of four plant species at a dew intensity of 0.1 mm, with the results shown in Figure 1. Among them, the *Tillandsia ionantha* had the fastest disappearance time at 10 min 51 s ± 0.57 s, while *O. sativa* was the slowest at 80 min 54 s ± 0.66 s. The order of leaf dew disappearance from fastest to slowest was: *Tillandsia ionantha* > *Oxalis triangularis* ‘Purpurea’ > *Epipremnum aureum* > *Oryza sativa*. Although dew disappearance time cannot directly distinguish between absorption and evaporation, it can still serve as a relative indicator of leaf water-use capacity among different plant species.

### 2.2. Proportion of Dew Absorbed by Plants

**Figure 2:**
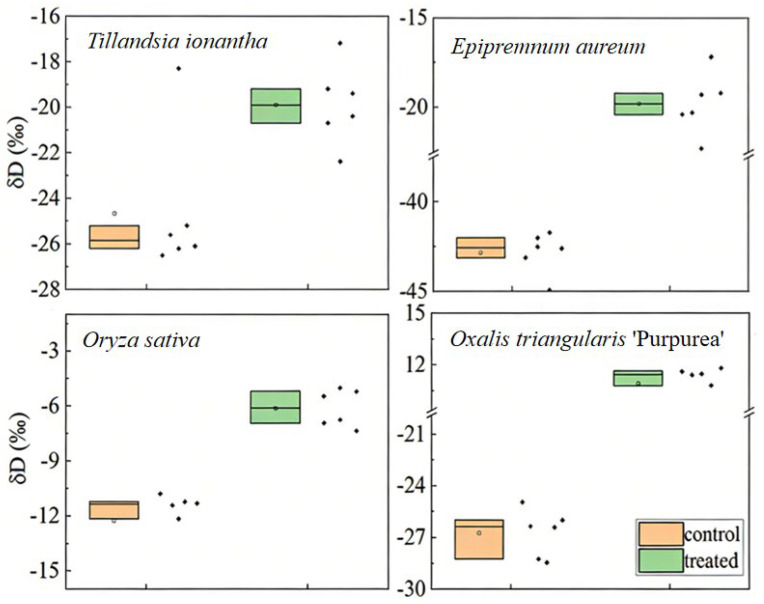
Changes in leaf water δD values of four plant species before and after the 0.1 mm dew treatment. Leaf water δD values after treatment with labeled artificial dew (&D = 946.852 ± 8.17‰) were significantly higher than those before labeling (*p* < 0.05). Box plots represent the δD values of different plant species (*n* = 6). Additional dew intensity treatments of 0.1 mm, 0.2 mm, and 0.3 mm were applied to *T. ionantha* and *E. aureum*. The results showed that the leaf water contents of *T. ionantha* under different dew intensities were 80.70 ± 0.59% at 0.1 mm, 64.45 ± 2.03% at 0.2 mm, and 51.71 ± 0.57% at 0.3 mm, respectively. In contrast, the leaf water contents of *E. aureum* were 6.02 ± 1.08% at 0.1 mm, 14.22 ± 2.13% at 0.20 mm, and 5.73 ± 3.85% at 0.3 mm (Table 1). Experimental calculations showed that the saturated leaf water contents of *T. ionantha* and *E. aureum* were 95.75% and 15.26%, respectively. Owing to its strong capacity for absorbing water vapor and its efficient use of atmospheric moisture, *T. ionantha* leaves reached near-saturation water content already under the 0.1 mm treatment, and even small amounts of dew were sufficient to trigger internal water movement. In *E. aureum*, the highest proportion of foliar water uptake occurred under the 0.2 mm treatment, with a leaf water content of 14.22 ± 2.13% and a water uptake proportion of 6.15 ± 3.12%, which was close to its saturated level.

**Figure 3:**
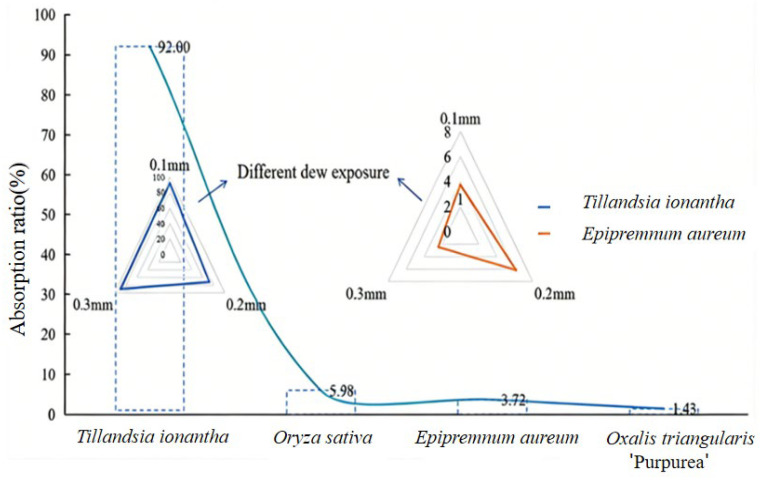
Proportion of dew absorbed by the leaves of four plant species under a dew intensity of 0.10 mm, and the proportion of dew absorbed by *Tillandsia ionantha* and *Epipremnum aureum* under different dew intensities. The line chart shows the proportion of dew uptake by plants at 0.1 mm dew intensity (*p* < 0.05). The triangular radar chart represents the absorption proportion of *Tillandsia ionantha* (blue) and *Epipremnum aureum* (orange) under different dew intensities (*n* = 6).

Under the 0.10 mm dew treatment, changes in δD values before and after isotopic labeling were compared among *T. ionantha*, *E. aureum*, *O. sativa*, and *O. triangularis* ‘Purpurea’. The results showed that the leaf water δD values of all four plant species increased significantly after labeling (*p* < 0.05) (Figure 2). The significantly higher δD values after labeling compared with those before labeling indicate that the plants absorbed water via foliar uptake, confirming that plants can directly take up dew via foliar uptake. Analysis of the proportion of dew absorbed by leaves among the four plant species showed significant interspecific differences (*p* < 0.05) (Figure 3), with the proportion of dew uptake ranked as follows: *T. ionantha* > *O. sativa* > *E. aureum* > *O. triangularis ‘Purpurea’*. Changes in δD values after simulated dew exposure in *T. ionantha*, *E. aureum*, *O. sativa*, and *O. triangularis* ‘Purpurea’ further verified the water uptake capacity of plants and confirmed the existence of different uptake pathways.

**Table 1:** Leaf Water Absorption Ratios of Plants under Different Dew Quantities.

Plant	Dew (mm)	0.1	0.2	0.3
*Tillandsia* *ionantha*	W (%)	80.70 ± 0.59	64.45 ± 2.03	51.71 ± 0.57
	Disappearance time	10′41″ ± 0.97″	18′15” ± 2.57″	25′01″ ± 5.37″
	*f*_a_ (%)	92 ± 3.71%	89.60 ± 2.43%	71.74 ± 5.10%
*Epipremnum aureum*	W (%)	6.02 ± 1.08	14.22 ± 2.13	5.73 ± 3.85
	Disappearance time	42′41″ ± 1.71″	46′41″ ± 3.51″	58′41″ ± 4.30″
	*f*_a_ (%)	3.72 ± 1.97%	6.15 ± 3.12%	2.45 ± 2.11%

### 2.3. Transport of Water After Foliar Dew Uptake in Plants 

**Figure 4:**
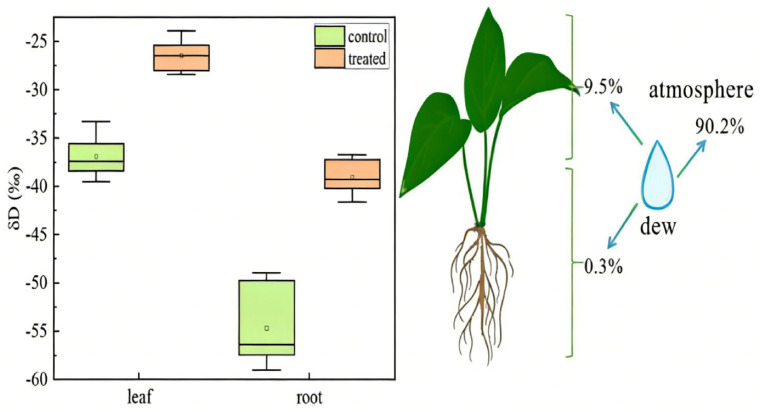
Comparison of δD values for *Epipremnum aureum* roots and stems. Orange represents the control group (no dew spraying), and green represents the labeled group (sprayed with artificial dew, &D = 946.852 ± 8.17‰). A total of 9.50% of the dew was absorbed by the leaves, 0.30% by the roots and stems, and the remaining dew evaporated into the atmosphere (*n* = 6).

In the isotope transport experiment conducted on *E. aureum*, the mean δD values of leaf water and root–stem water were −36.93 ± 2.03‰ and −54.65 ± 3.80‰, respectively. After spraying labeled water at the same intensity, the δD values of leaf water and root–stem water changed to −26.43 ± 1.57‰ and 39.03 ± 1.71‰, respectively, indicating that water absorbed through the leaves and stems could be transported to the roots, and vice versa. The proportions of dew absorbed in the leaf and root–stem regions were 9.50% and 0.30% (Figure 4), respectively.

After labeling, the increase in leaf water δD (10.50‰) was substantially smaller than that in root–stem water δD (approximately 93.68‰), indicating a substantial spatial redistribution of the labeled water within the plant. Compared with the pre-labeling values, root–stem water δD changed from being significantly lower than leaf water to significantly higher, suggesting that the labeled water was rapidly transported from the leaves and stems into the vascular system and subsequently to the roots.

## 3. Discussion

### 3.1. Factors Affecting Differences in Dew Uptake Among Leaves of Different Plant Species

Under the same experimental conditions, dew retention time, i.e., the disappearance time, represents the physical process of water remaining on the leaf surface and is mainly influenced by evaporation rate, surface roughness, and hydrophobicity [18]. In contrast, dew absorption reflects the physiological process of water entering the leaf interior, which is primarily influenced by the coordinated interaction of external leaf physical traits and the driving force of internal leaf water potential [19,20]. Since these two processes are controlled by different mechanisms, situations may arise in which “dew disappears quickly but absorption is high” (e.g., *Tillandsia ionantha*), or “dew remains on the leaf for a long time but the absorption proportion is not necessarily the highest” (e.g., *Oryza sativa*). In other words, the duration of dew presence on the leaf surface does not equate to effective absorption time. If the leaf possesses highly efficient absorption structures, dew can be rapidly utilized even during short retention periods; conversely, even if dew persists for a long time, it cannot be effectively converted into plant-available water if efficient entry pathways are lacking [21].

The leaves of *T. ionantha* are densely covered with scale-like trichomes, which not only help the plant rapidly absorb dew but also significantly increase the leaf surface area-to-volume ratio. This structural adaptation allows moisture to spread more easily into thin films and accelerates evaporation, resulting in the fastest disappearance of dew [22]. Research has shown that the evaporation time of water droplets varies depending on different leaf surface characteristics (such as smooth vs. non-smooth leaf surfaces). Smooth leaf surfaces tend to have a greater number of small droplets, but the overall coverage area and retention effectiveness are inferior to those on rough leaf surfaces. Smooth leaf surfaces are less conducive to maintaining prolonged droplet residence, thereby promoting evaporation and dew disappearance [23,24]. Studies have shown that under light conditions, small and smooth leaves tend to warm up relatively quickly, thereby increasing the evaporation rate [25]. This may explain why *Oxalis triangularis* ‘Purpurea’, with its small, smooth leaves and thin waxy layer, heats up relatively quickly under light, leading to a high evaporation rate and faster dissipation of dew. *Epipremnum aureum* leaves have a relatively thick cuticle and a smooth surface, which allows water droplets to remain as larger droplets, resulting in a relatively slower evaporation rate from the leaves [26]. *O. sativa* leaves possess prominent longitudinal groove structures and strong surface hydrophobicity, causing water droplets to converge along the veins and form stable beads or slowly flow along the leaf surface. This reduces the evaporation rate per unit area and may explain why water retention time on rice leaves is the longest [27].

**Figure 5:**
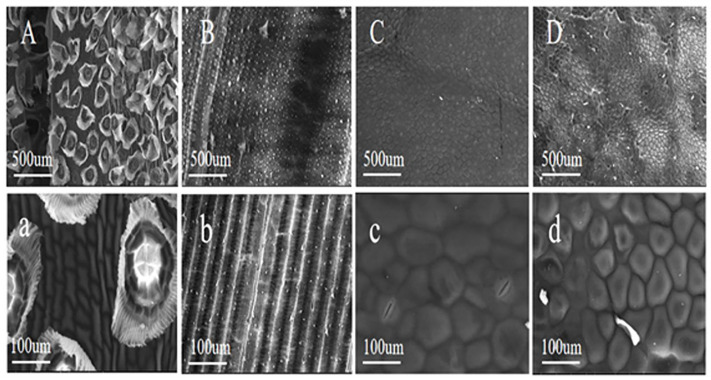
Microscopic structures of leaves of four plant species. Images (**a**–**d**): *Tillandsia ionantha* (**a**), *Oryza sativa* (**b**), *Epipremnum aureum* (**c**), and *Oxalis triangularis ‘Purpurea’* (**d**). Scale bars: 100 µm. Images (**A**–**D**): *Tillandsia ionantha* (**A**), *Oryza sativa* (**B**), *Epipremnum aureum* (**C**), and *Oxalis triangularis* ‘Purpurea’ (**D**). Scale bars: 500 µm.

After undergoing a period of uniform cultivation, which eliminated the potential effects of transplanting stress and differences in the original environment on plant water metabolism and leaf structural function, the water potential within the leaves is expected to be relatively stable. The proportion of dew absorbed by the leaves of the four plant species is therefore primarily determined by leaf surface morphology and microstructural traits (Figure 5), as well as the resulting wettability and effectiveness of water entry pathways. According to previous studies, the main microscopic factors causing differences in leaf water absorption include trichomes, cuticle thickness, wax layer continuity, and stomata, among others [7,12]. *T. ionantha* has evolved a physiological strategy primarily based on foliar water uptake, exhibiting a strong dependence on and high efficiency in utilizing water on the leaf surface. Its leaf surface is densely covered with shield-shaped trichomes, which not only efficiently capture liquid water but also enable rapid water absorption through the thin-walled cells at the base of the trichomes and associated hydrophilic pathways [28,29]. In addition, the leaf surface of *T. ionantha* also features honeycomb-like pores with diameters of approximately 200–500 µm, which are hexagonal or polygonal in shape, with slightly curled or folded edges. This porous structure is similar to other materials with strong water absorption and retention capabilities, such as certain biological tissues (e.g., wood or bone) or synthetic materials (e.g., porous ceramics or polymer foams), thereby facilitating dew absorption by *T. ionantha* (Figure 5A,a). For example, the cactus Opuntia stricta has evolved exceptional water absorption and storage capabilities to adapt to arid environments. Its spiny leaves possess barbs and groove structures, enabling coordinated water uptake between the leaves (spines) and stems, thereby enhancing the collection and utilization of dew [30]. Tillandsia usneoides, a congener of the *T. ionantha* used in this study, exhibits similar traits, and its dew absorption rate is higher compared to other species [31]. *O. sativa* leaves possess pronounced longitudinal groove structures and a silicified epidermis, which can effectively retain water droplets and guide water along the veins, thereby increasing the contact area between water and the epidermis and enhancing droplet stability on the leaf surface. Meanwhile, *O. sativa* stomata are mostly distributed along the side walls of the grooves, where local water films may form under high humidity conditions, promoting water entry into the leaf through the areas surrounding stomata or through cuticular micropores. In addition, *O. sativa* typically grows in high-humidity or flooded environments and thus shows a certain degree of adaptation to foliar water uptake. Although *O. sativa* leaves have a relatively thick wax layer, other structural and functional traits that are more decisive for dew uptake may override this barrier [32], resulting in a higher proportion of dew absorption than that of *E. aureum* and *O. triangularis* ‘Purpurea’. Similarly, leaves of Hippophae rhamnoides in arid and cold regions possess a relatively thick waxy cuticle; however, due to the presence of stellate trichomes and a higher density of stomata on the abaxial leaf surface, they are able to effectively utilize dew and fog water [33].

The leaves of *E. aureum* have a relatively thick cuticle and a smooth surface, with a well-developed, continuous wax layer. These features may limit water diffusion through the cuticle into the leaf interior, resulting in a relatively low overall water absorption [34]. In addition, *E. aureum* primarily relies on root water uptake, and its leaves have not evolved specialized structures for directly absorbing liquid water; therefore, foliar water uptake is clearly constrained by leaf structure [35,36]. For example, *Ulmus* leaves have a thick cuticle with a continuous, highly hydrophobic epicuticular wax layer. The leaf surface is relatively flat and lacks microstructures capable of efficiently capturing water, so water uptake primarily relies on root absorption and transport through the xylem [33]. The needle surfaces of *Abies bacteata* are covered with a thick cuticle and dense epicuticular wax, rendering them highly hydrophobic and effectively preventing liquid water from spreading across the leaf surface or penetrating into the leaf interior. Stomata in fir needles are typically sunken in stomatal pits and are mostly located on the abaxial surface. Their structural design primarily serves to reduce transpiration loss rather than to facilitate the entry of liquid water through the stomata, and therefore contributes little to dew absorption [37]. The leaf surfaces of *O. triangularis* are covered with abundant hydrophobic hairs, which significantly reduce the direct contact area between water droplets and epidermal cells, causing droplets to remain more spherical and either roll off or evaporate quickly, thereby limiting opportunities for water to enter the leaf. In addition, the cuticle strongly impedes water diffusion, and as a typical shallow-rooted herbaceous species, this plant relies minimally on foliar water uptake and lacks specialized structures for absorbing water through the leaf surface. Consequently, it exhibits the lowest dew absorption ratio among the studied species. For example, the abaxial surface of *Quercus ilex* leaves is covered with dense hairs and associated microstructures, rendering it highly hydrophobic and water-repellent. This prevents water droplets from wetting the leaf surface and entering the leaf interior. Many leaf surface traits, such as hydrophobic hairs and epicuticular wax crystals, confer water-repellent properties; after rainfall, dew, or fog events, this hydrophobicity causes droplets to remain spherical with large contact angles, reducing leaf surface wetting and foliar water uptake [38].

### 3.2. Response of Foliar Water Uptake to Varying Dew Intensities in Plants

The results of this study indicate that both *T. ionantha* and *E. aureum* exhibit clearly nonlinear foliar water uptake responses to varying dew intensities, with distinct species-specific patterns. *T. ionantha* showed a high leaf water content (80.70%) even under low dew intensity (0.1 mm), approaching its saturated water content (95.75%), indicating that its foliar water uptake structures are highly sensitive to atmospheric moisture, and even a small amount of dew can rapidly trigger effective water absorption and internal redistribution. However, as dew intensity increased to 0.2 mm and 0.3 mm, leaf water content gradually declined. This pattern may result from a combination of stomatal regulation and passive saturation effects of the leaf water uptake structures under high humidity or elevated external water availability, leading to a negative feedback mechanism characterized by reduced absorption efficiency at higher dew intensities [39]. For example, in mangrove species with strong leaf water uptake capacity, leaf surface microstructures (such as trichomes and surface wettability) play a crucial role in regulating water absorption [40]. Once sufficient water has been absorbed under low-intensity wetting, additional water inputs no longer enhance uptake and may even inhibit further absorption. This regulatory mechanism helps prevent excessive water uptake that could lead to cellular turgor imbalance or metabolic disturbance, and reflects a water-use strategy in epiphytic plants that combines rapid responses to short-term water pulses with self-protection mechanisms [20].

Compared with this, *E. aureum* exhibited a hump-shaped response across different dew intensities: the absorption ratio was low under the 0.1 mm treatment, increased to a maximum at 0.2 mm, where leaf water content approached saturation (14.22%, compared with a saturated value of 15.26%), and then declined again under the 0.3 mm treatment. This may be because foliar water uptake in *E. aureum* is constrained by cuticular permeability and the degree of stomatal opening; only after a certain water film thickness or a sufficiently long wetting duration is reached can water effectively enter the internal leaf tissues [41]. However, when the dew amount further increases, an excessively thick water film may inhibit stomatal gas exchange and induce partial stomatal closure, thereby further limiting water uptake. In addition, excessive water can increase leaf surface runoff and evaporative losses, reducing the proportion of the input water that is actually absorbed [42].

### 3.3. Distribution and Internal Transport of Dew After Foliar Absorption in Plants

Water absorbed by the leaves and stems of *E. aureum* can rapidly enter the vascular system and be transported to the roots, and the labeled water undergoes clear spatial redistribution within the plant. This observation is consistent with previous studies showing that, in Japanese cedar seedlings, water absorbed by the leaves is not only used to maintain leaf water potential but is also transported into internal tissues to participate in biosynthesis, thereby influencing the hydrogen and oxygen isotope composition of plant water. After absorption, water is redistributed between leaves and vascular tissues through water potential gradients as well as diffusion and mass flow (percolation) mechanisms [35]. Taking tomato as an example, the transport rate of internal leaf water (mesophyll water) under different water supply conditions and its responses were measured. The results showed that water within the leaf passes through the intracellular pools of mesophyll cells and then enters the vascular bundles or surrounding tissues [43]. The δD of root–stem water shifted from being significantly lower than that of leaf water to higher than that of leaf water, further indicating that the labeled water absorbed by the leaves was efficiently transported to the roots within a short time. In addition, the proportions of dew absorbed by the leaves and by the roots–stems were 9.50% and 0.30%, respectively, indicating that foliar water uptake is the primary pathway for water supplementation in the plant. Roots and stems are also pathways for dew uptake, but their direct contribution to dew absorption is relatively limited [13]. These results reveal the rapid internal redistribution of water among different organs in *E. aureum*, suggesting that in natural environments, plants can adapt to intermittent water availability through foliar water uptake and internal water regulation mechanisms. Studies have found that in arid sandy-land shrubs, water absorbed by the leaves is transported along the vascular bundles to the stems, helping to improve the plant’s water balance [11]. In the redwood forest environment of California, 80.00% of plant species possess the ability for foliar water uptake. Leaf water absorption enhances the overall plant water potential and reduces nocturnal water loss, thereby facilitating more efficient replenishment of water reserves in the roots [9]. After absorbing water, the branches and leaves of *Calligonum mongolicum* generate a water potential gradient that drives water movement downward from the leaves and branches to lower plant parts, increasing the water potential throughout the plant tissues. A higher water potential supports elevated photosynthetic rates and stomatal conductance, which can also indirectly improve root water use and physiological function [44].

Although non-precipitation water inputs have received growing scholarly attention, research explicitly addressing plant dew uptake remains scarce. The composition and timing of water on different types of leaf surfaces—such as rain, fog, dew, and irrigation water—may all have varying effects. According to an integration of multiple global plant databases, approximately 369,000 vascular plant species have been validly described worldwide, highlighting the remarkable diversity of plant forms and functional traits [45]. For this study, the three experimental parts only compared dew absorption under different conditions for the four selected plant species, and the absorption–transport experiment was conducted solely on the relatively ideal species, *E. aureum*. This scope is somewhat limited. Future scientific investigations should expand the range of plant species to further explore foliar dew absorption, thereby enhancing our understanding of the characteristics and mechanisms of plant dew utilization.

## 4. Materials and Methods

### 4.1. Study Site and Plant Material Selection

**Figure 6:**
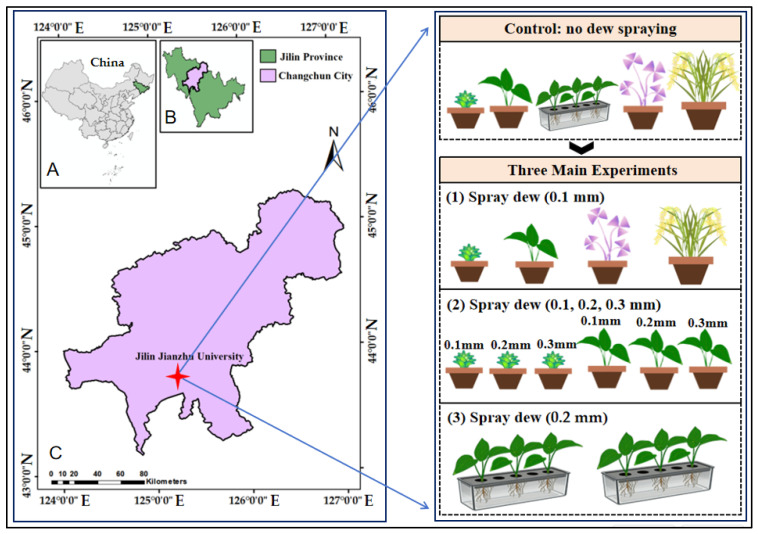
Location of the study area (Jilin Jianzhu University, Nanguan District, Changchun, Jilin Province, China; 43°50′ N, 125°20′ E). (**A**): shows China. (**B**): shows Jilin Province. (**C**): shows Changchun City; the star indicates the study site. (**1**): all four species treated with 0.1 mm distilled water and labeled dew, respectively (*n* = 6). (**2**): *Tillandsia ionantha* and *Epipremnum aureum* treated with 0.1, 0.2, and 0.3 mm distilled water and labeled dew, respectively (*n* = 6). (**3**): hydroponic isotope transport experiments on *Epipremnum aureum* (two trays, three plants per tray; *n* = 6).

This study was conducted in a controlled-environment greenhouse laboratory at Jilin University of Architecture, Nanguan District, Changchun, Jilin Province (43°50′ N, 125°20′ E) (Figure 6). To investigate the utilization of dew by different plants and to measure and monitor their various morphological and physiological traits, four temperate plant species with distinct characteristics were selected: *Tillandsia ionantha*, *Epipremnum aureum*, *Oryza sativa*, and *Oxalis triangularis* ‘Purpurea’. These selected plants each possess unique leaf surface structures and plant traits, as summarized in Table 2.

The four plant species exhibit distinct differences in atmospheric water use, leaf structure and morphology, root and stem traits, as well as physiological characteristics and adaptability, allowing a multidimensional investigation of their dew absorption capacities. Among them, *T*. *ionantha* is a bulbous epiphyte that can grow epiphytically without soil, primarily absorbing water through its leaves. Its leaf surface is covered with scales, which vary in shape, arrangement, and density. The leaf cells are nearly square, with slightly undulating cell walls and variable cell sizes, eatures associated with high water absorption capacity [46,47]. *E*. *aureum* is a succulent plant that can absorb water through both its roots and leaves, though it primarily relies on the soil. Its leaves are elongated and elliptical, dark green or with yellow streaks, and are covered with numerous stomata that may facilitate the uptake of atmospheric moisture. Additionally, its roots have abundant root hairs, facilitating water uptake from the soil [48]. *O*. *sativa* is an annual herb and one of the world’s most important food crops, showing a high dew condensation rate among crops in the Sanjiang Plain, China [49]. Its leaves have trichomes and stomata, enabling the absorption of atmospheric moisture in aquatic environments, while its hollow stems store water to help withstand drought. Trichomes and stomata on the leaf underside further facilitate the uptake of atmospheric water [50]. *O*. *triangularis* ‘Purpurea’ has leaves that are inverted-triangular or broad-arrow shaped, with wavy margins, a purplish-red color, and sparse white pubescence. This perennial herb exhibits nyctinastic behavior, opening during the day and closing at night. The triangular leaf shape aids in light capture and enhances wind resistance. Its rhizomes are spindle- or oblong-shaped, storing water and nutrients, which allows the plant to survive under drought conditions [51,52].

### 4.2. Experimental Design

#### 4.2.1. Experimental Pre-Treatment

This experiment included an isotope-labeled treatment and a control. The labeled treatment used heavy water (D_2_O, 99.9 atom% D; Sigma-Aldrich, St. Louis, MO, USA) to simulate dew, which was diluted with distilled water at a ratio of 1:15,000 to prepare the labeled solution, with a δD value of 946.852 ± 8.17‰. During the foliar heavy water uptake experiments under different dew-intensity conditions, the diluted heavy water was uniformly applied to the leaf surfaces according to the above ratio. The prepared heavy water was loaded into a stable spraying system, with 0.85 mL delivered per spray. Based on the leaf area of the four plant species, the total volume of simulated dew to be applied was calculated, and the number of sprays was rounded to the nearest integer according to the target water amount. Leaf area of each plant was measured using the grid-counting method, and dew intensity was converted to the target sprayed dew volume using the standard formula [53]:

(1) $$W_{l}=\frac{I\times S_{leaf}}{10}$$

where *W_l_* is the dew quantity in grams (g); *I* is the dew intensity in millimeters (mm); and *S_leaf_* is the leaf area in square centimeters (cm^2^). The number 10 is a conversion factor used to adjust the units.

To approximate favorable growth conditions for temperate plants, the greenhouse temperature was maintained at approximately 23 °C, with relative humidity controlled at 80%. Light was provided by full-spectrum lamps with an intensity of about 300 μmol m^−2^ s^−1^, approximating natural daylight [54]. All four experimental plant species were cultivated in pots of the same size. The rootless *T. ionantha* received no special treatment and was placed directly in pots with internal supports. *E. aureum* and *O. triangularis* ‘Purpurea’ were planted in pots containing the same soil conditions. To prevent sprayed dew from dripping into the soil and being absorbed by roots, the pots were sealed with aluminum foil, leaving only the shoots exposed, thereby reducing potential experimental error. To simulate normal growing conditions, *O. sativa* was placed in pots filled with tap water that had been left at room temperature for 8 h, mimicking flooded paddy conditions, and these pots were also sealed. To minimize potential effects of prior growth conditions, all plants were acclimated in the greenhouse for seven days before the experiment to reduce transplant shock and environmental differences, allowing their physiological status to stabilize before measurements were conducted [55].

#### 4.2.2. Overview of the Three Main Experiments

**Figure 7:**
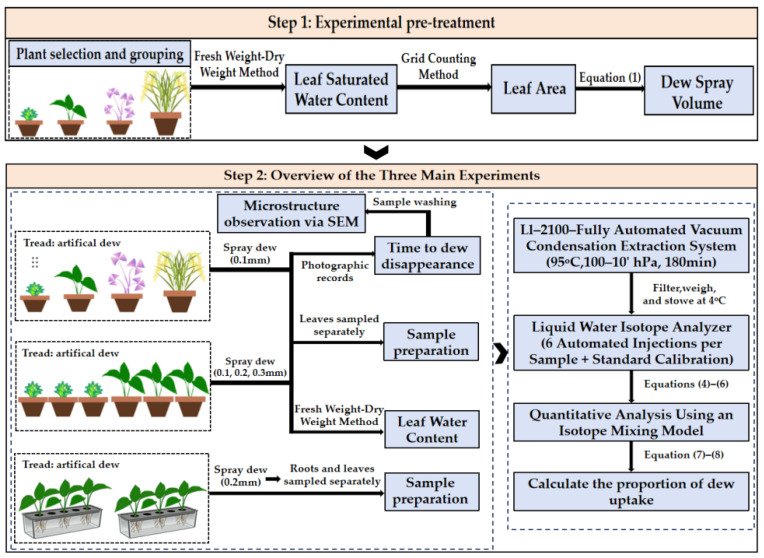
Experimental workflow for plant dew absorption and stable isotope analysis.

This study was divided into three main parts, and different dew treatments were applied to four plant species (Figure 7), with the pretreatment and experimental design workflow shown in Figure 7. Previous field measurements of dew have indicated that daily dew amounts are commonly around 0.1 mm, and annual daily dew levels typically range from 0.1 to 0.3 mm/day [56]. In the first part of this study, the aim was to investigate the dew absorption of different plant types. All four species were subjected to a 0.1 mm dew treatment. After uniformly spraying the simulated dew onto the leaves of each plant group, the surface water was recorded using a high-definition camera every 10 min. When only tiny droplets remained on the leaf surface, images were taken every 2 min. Dew disappearance time was recorded when no visible water droplets or films remained on the leaves.

For the four selected plant species, *O. sativa* primarily absorbs water through its roots, and its specialized leaf structure tends to cause dew to run off, while the small leaves of *O. triangularis* ‘Purpurea’ present a similar concern. Therefore, in the second part of this study, to investigate dew absorption under different dew intensities, we selected *T. ionantha* and *E. aureum*, which have clearly measurable and significant absorption capacities, and applied dew treatments of 0.1 mm, 0.2 mm, and 0.3 mm. Prior to the experiment, plants of *T. ionantha* and *E. aureum* cultured under the same conditions were chosen to measure leaf saturated water content. For both experimental parts, six replicates were set for each of the control and labeled groups.

Among the four selected plant species, *E. aureum* has a well-developed vascular system and clearly differentiated root–stem–leaf structures, providing defined pathways for water transport. This facilitates the accurate tracing of water movement and distribution among different organs using isotopic labeling. Additionally, its roots develop rapidly and maintain stable morphology, making it convenient to perform separate sampling of roots, stems, and leaves under controlled conditions, thereby enhancing the reliability and reproducibility of the experimental results. Therefore, this experiment focused on *E. aureum* as the study species, conducting isotope transport experiments on its roots, stems, and leaves. Six cuttings of *E. aureum* with similar leaf size and growth vigor were selected for hydroponic culture. Prior to the experiment, the cuttings underwent a four-week rooting and acclimation period. During the rooting stage, the water was replaced every three days, and rooting required approximately ten days; thereafter, water was changed every seven days. Heavy water was sprayed onto the leaves of *E. aureum*, and for both the experimental and control groups, roots and stems were separated and processed using vacuum freeze extraction, with each step repeated three times [57].

### 4.3. Sample Collection and Analysis

The leaf water content (*W*_r_) of the four plant species, as well as the saturated water content (*W*_s_) of *T.* and *E. aureum* leaves, was determied using the fresh mass–dry mass method. For the relative water content of the four plants after dew treatment, mature functional leaves that are robust, free of disease spots, undamaged, and of similar size should be selected as sampling objects. After treatment, immediately use a sterile knife to cut the leaves, quickly wipe off any residual water on the surface with moist filter paper to avoid interference from water droplets during fresh weight measurement, and immediately weigh to obtain the fresh weight (*W*_1_). Subsequently, the samples were dried in a constant-temperature oven at 80 °C to constant weight (for 48 h), and after cooling, the dry mass (*W*_2_) was recorded [58]. The calculation method is as follows:

(2) $$W_{r}=\frac{W_{1}-W_{2}}{W_{1}}\times 100\%$$

where *W*_1_ is the leaf relative water content (%), *W*_1_ is the mass measured immediately after cutting (g), and *W*_2_ is the mass measured after drying at 80 °C to constant weight (g).

For determining the saturated water content of *T. ionantha* and *E. aureum* leaves, leaves were selected and handled in the same manner, then gently rinsed with deionized water to remove surface impurities. After blotting off free surface water with filter paper, the initial fresh mass (*W*_3_) was measured. The leaves were then completely immersed in deionized water and incubated for 24 h at room temperature in the dark to allow full water uptake and reach tissue water saturation. After removal, surface water was gently blotted off with filter paper, and the saturated fresh mass (*W*_4_) was measured, followed by the same drying procedure described above [58]. The calculation method is as follows:

(3) $$W_{s}=\frac{W_{3}-W_{4}}{W_{3}}\times 100\%$$

where *W*_s_ is the leaf relative water content (%), *W*_3_ is the mass measured immediately after the leaves were completely immersed in deionized water and incubated for 24 h at room temperature in the dark to allow full water uptake and reach tissue water saturation(g), and *W*_4_ is the mass measured after drying at 80 °C to constant weight (g).

For isotope analysis, leaf tissue samples were excised with a sterile blade from 0.50–2.00 cm inward from the point of tracer application to avoid direct contamination by the tracer and ensure that the heavy water had fully penetrated the internal plant tissues. For the isotope transport experiment conducted on *E. aureum* roots, stems, and leaves, the plants were immediately removed from the hydroponic containers after the sprayed simulated dew had disappeared. While maintaining physiological stability, leaves and roots were carefully separated with a clean sterile blade to prevent cross-contamination, and residual surface water on the leaves was quickly removed. The freshly collected plant tissue samples were then transferred to glass containers, and water was extracted using a low-temperature vacuum distillation method. During the experiment using the fully automated vacuum condensation extraction system (LI-2100, LICA United Technology Limited, Beijing, China), the distillation conditions were set at 95 °C and a vacuum pressure of 100–10^−1^ hPa for a duration of 180 min. All collected water samples were filtered through a 45 μm pore-size membrane and then stored in pre-weighed 0.5 mL glass vials (*W*_a_). The combined weight of the sample and vial was recorded again (*W*_b_), and the samples were stored at 4 °C for subsequent isotope analysis. The isotopic composition was determined using a liquid water stable isotope analyzer (LGR, LWIA-30d, San Jose, CA, USA) with a measurement precision of 0.6‰. To improve analytical accuracy, two isotope standards (δD = −51.0 ± 0.5‰ and −9.5 ± 0.5‰) were introduced before and after each sample analysis. Each sample and standard was injected six times to ensure the stability and reliability of the isotope measurements. For each injection, a 1.0 μL syringe was used to withdraw 0.8 μL of sample, and the injection procedure was completed according to the instrument instructions. After each sample was measured and before extracting the next sample, the syringe was thoroughly rinsed with deionized water to remove residual sample and minimize analytical errors. The formula for calculating the amount of dew absorbed by plants using the isotope mixing model is as follows [12,59]:

(4) $$\delta_{P}=f_{A}\times \delta_{A}+f_{B}\times \delta_{B}$$

(5) $$1=f_{A}+f_{B}$$

(6) $$f_{A}=\frac{\delta_{P}-\delta_{B}}{\delta_{A}-\delta_{B}}$$

where *δ*_P_ represents the δD value of leaf water after labeling, *δ*_A_ represents the δD value of simulated dew, and *δ*_B_ is the δD value of leaf water before spraying the labeled water; *f*_A_ represents the proportion of simulated dew in the plant leaf water, and *f*_B_ represents the proportion of leaf water before labeling in the plant leaf water.

Then, the proportion of dew absorbed by the plant during the evaporation process is calculated using the following formula [59]:

(7) $$f_{a}=\frac{W_{e}\times f_{A}\times 10}{S_{\mathrm{leaf}}\times I\times n}$$

*W*_e_ = *W*_b_ − *W*_a_ (8)

where *f*_a_ is the proportion of dew absorbed by the plant during the evaporation process (%); *W*_e_ is the weight of leaf water obtained by vacuum extraction (g); *f*_A_ is the percentage of dew in the leaf water (%); *S_leaf_* is the area of a single leaf (cm^2^); *I* is the dew intensity (mm); *n* is the number of leaves sampled after dew evaporation, and 10 is a unit conversion factor. *W*_b_ is the mass of the glass vial (g), *W*_a_ is Mass of the glass vial plus the extracted water sample (g).

### 4.4. Structural Observation with Scanning Electron Microscope (SEM)

Fresh leaves from the experimental plants were selected and cut into appropriately sized pieces for observation. The leaves were ultrasonically cleaned for 2 h to remove surface particles and other impurities, eliminating potential interference with the microscopic structural examination [60]. After drying, the leaves were observed using a field emission scanning electron microscope (SEM, FEI Quanta 450, FEI Company, Hillsboro, OR, USA) to examine microstructures such as trichomes, wax types, stomata, and surface ridges and folds.

### 4.5. Data Analysis

The raw data were organized and preprocessed using Microsoft Excel, followed by statistical analysis with IBM SPSS Statistics 26. Data visualization was performed using OriginPro 2025 to clearly illustrate differences and trends among the different treatment groups.

## 5. Conclusions

The four plant species selected in this study all possess the ability to absorb dew through their leaves, but the absorption efficiency varies significantly and is closely related to leaf microstructure and surface physical traits. Under a dew intensity of 0.1 mm, *T. ionantha* exhibited the highest dew absorption rate at 92%, facilitated by its dense shield-shaped scales and trichomes, whereas *O. triangularis* ‘Purpurea’, whose leaves are covered with hydrophobic hairs, showed the lowest absorption rate at only 1.43%. Across different dew intensities, the absorption rate of *T. ionantha* decreased with increasing dew (0.1 mm: 92%; 0.2 mm: 89.60%; 0.3 mm: 71.74%), suggesting a high sensitivity to atmospheric water and a potential passive saturation effect. In contrast, *E. aureum* exhibited its highest observed leaf water absorption (6.15%) at 0.2 mm, suggesting that its foliar water uptake is likely limited by cuticle permeability and stomatal regulation. Water absorbed through the leaves can be transported via the vascular system to the roots; in *E. aureum*, the absorption proportions for leaves and roots were 9.50% and 0.3%, respectively, indicating that leaf-absorbed dew can contribute to root water content and is an important pathway for plant water supplementation. Overall, dew, as a non-precipitation water source, plays a crucial role in maintaining plant water balance, and its utilization efficiency is jointly regulated by leaf structure, micro-morphology, and dew intensity. This study provides quantitative evidence for the ecological adaptation and water use strategies of different plants and offers important insights into plant adaptation to environmental water availability.

## Figures and Tables

**Table 2:** Types and Leaf Surface Characteristics of Selected Plants.

Plant Species	Family	Genus	Plant Height (cm)	Blade Shape	Leaf Area (cm^2^)	Waxy Layer	Leaf Surface Texture
*Tillandsia* *ionantha*	Bromeliaceae	Tillandsia	15–30	Long oval	10–15	Thin	Rough
*Epipremnum aureum*	Araceae	Epipremnum	30–100	Heart-shaped	30–50	Thick	Smooth
*Oryza sativa*	Poaceae	Oryza	60–120	Long strip	60–120	Thick	Parallel
*Oxalis triangularis ‘Purpurea’*	Oxalidaceae	Oxalis	10–30	Inverted triangle	15–30	Thin	Smooth

## Data Availability

The data presented in this study are available on request from the corresponding author.
